# Treatment of Emergencies

**Published:** 1887-07-23

**Authors:** 


### Till the Doctor Comes.

Inquiries and Communications for this column should be addressed "Physician," care of the Editor of THE HOSPITAL.]

### XXII.—TREATMENT OF EMERGENCIES.

(*Continued.*)

*Spitting of Blood.*—Keep the patient raised in bed; let him avoid coughing as much as possible; give him ice to suck, and iced milk to drink; keep the room cool; do not give anything hot; if the spitting is profuse, put an ice-bag to his chest; let him keep perfectly quiet, and not exert himself in any way. He must not even get out of bed on any pretence.

*Vomiting of Blood.*—The same rules apply, but instead of putting an ice-bag to the chest, apply a mustard plaster over the stomach.

In all these cases do not be in a hurry to give stimulants; a little faintness does good, as it tends to restrain the bleeding. See also that there is nothing tight about the limbs or neck that may interfere with the circulation.

*Suffocation.*—An interruption of the function of respiration, which, sufficiently prolonged, causes death. Under this head we may conveniently include not only drowning, strangling, and hanging, but also those cases in which food gets into the windpipe and obstructs the passage, and those in which deleterious gases are breathed to a dangerous degree, as the gases from burning charcoal, escape of ordinary gas, etc. When the respiration is totally obstructed, all external movements cease within five minutes, and the heart within ten minutes. Restoration is possible as long as the heart continues to beat, but some cases of drowning have been restored after a much longer period (half-an-hour), it being then probable that the person has fainted at the moment of immersion. Even in apparently hopeless cases, therefore, all means should be tried for some considerable time. The treatment of persons apparently drowned may be taken as a type, the differences in other forms of suffocation being subsequently noticed.

*Drowning.*—Raise the patient, and hold the head downwards for a moment, to allow any water to escape; cleanse mouth and nostrils; open the mouth; keep the tongue forward; loosen all tight clothing. Place him on his back, and keep head and shoulders slightly raised. Grasp his arms just above the elbows, and draw them gently and steadily upwards till they meet above the head (this is for the purpose of drawing air into the lungs); keep the arms in that position for two seconds, then turn them down, and press them gently and firmly for two seconds against the sides of the chest (for the purpose of pressing air out of the lungs). Repeat these measures alternately fifteen times in a minute, till the patient is seen to make a voluntary effort to breathe. (This is known as Sylvester's method of performing artificial respiration, and is the best.) Whilst this is proceeding, others may be engaged in other ways, as in removing wet clothing, and wrapping him in blankets or dry clothes. Putting ammonia to the nose, tickling the back of the throat with a feather, and slapping the surface of the face and chest, are all useful in tending to provoke respiration. After breathing has been restored, it is necessary to induce warmth and circulation by applying hot flannels, bottles, etc., and by rubbing the limbs upwards firmly and quickly. Lastly, when he can swallow, small quantities of hot tea, wine and water, etc., may be given.

*Hanging and Strangling.*—Cut the patient down, and commence artificial respiration as above.

*Suffocation from Charcoal Fumes.*—Remove the person at once into the fresh air, and commence Sylvester's method of artificial respiration; but it is doubtful whether this will be of much service, as carbonic oxide is the most poisonous agent in these cases, and acts as a blood poison. Nothing else can be done, however, except by a medical man. Great care should be taken not to sleep in a room where there is no flue for the escape of fumes of burning charcoal, or into which there is a leakage from a stove-pipe.

*Suffocation from Carbonic Acid.*—This is the gas that is known as the choke-damp of mines. It also accumulates in the process of brewing over vats of fermenting beer, and is given off from limekilns, being met with also in old wells, volcanic grottoes, etc. Treatment in all these cases is removal into a pure atmosphere, and performance of artificial respiration. It is this gas which causes the headache, sense of oppression, and drowsiness felt in badly ventilated rooms, especially where much gas is being burnt.

*Poisoning from Escape of Gas.*—Carbonic oxide is such an active poison, that probably it is the chief cause of death in these cases, and the same treatment may be applied as from that poison.

(*To be continued.*)

